# Severe disease exacerbation after mRNA COVID-19 vaccination unmasks suspected multiple sclerosis as neuromyelitis optica spectrum disorder: a case report

**DOI:** 10.1186/s12883-022-02698-y

**Published:** 2022-05-18

**Authors:** Lisa Lohmann, Felix Glaser, Gabriel Möddel, Jan D. Lünemann, Heinz Wiendl, Luisa Klotz

**Affiliations:** Department of Neurology with Institute of Translational Neurology, University Hospital Münster, Albert-Schweitzer-Campus 1, 48149 Münster, Germany

**Keywords:** COVID-19, Vaccination, MS, NMOSD, Case report

## Abstract

**Background:**

Since the beginning of the COVID-19 pandemic and development of new vaccines, the issue of post-vaccination exacerbation or manifestation of demyelinating central nervous system (CNS) disorders has gained increasing attention.

**Case presentation:**

We present a case of a 68-year-old woman previously diagnosed with multiple sclerosis (MS) since the 1980s who suffered a rapidly progressive severe sensorimotor paraparesis with loss of bladder and bowel control due to an acute longitudinal extensive transverse myelitis (LETM) after immunization with the mRNA Pfizer–BioNTech COVID-19 vaccine. Detection of Aquaporin-4-antibodies (AQP4) in both serum and CSF led to diagnosis of AQP4-antibody positive neuromyelitis optica spectrum disorder (NMOSD). Treatment with intravenous corticosteroids and plasmapheresis led to a slight improvement of the patient’s symptoms.

**Conclusions:**

Pathogenic mechanisms of post-vaccination occurrence of NMOSD are still unknown. However, cases like this should make aware of rare neurological disorders manifesting after vaccination and potentially contribute to improvement of management of vaccinating patients with inflammatory CNS disorders in the future. So far two cases of AQP4-antibody positive NMOSD have been reported in association with viral vector COVID-19 vaccines. To our knowledge, we report the first case of AQP4-antibody positive NMOSD after immunization with an mRNA COVID-19-vaccine.

## Background

Post-vaccination exacerbation or manifestation of inflammatory central nervous system (CNS) disorders is a rare phenomenon that has been reported in association with few types of vaccinations (e.g. vaccination against human papilloma virus (HPV), influenza, tetanus) [1]. However potentially underlying mechanisms have not yet been fully understood and assumption of causality between vaccination and disease exacerbation is still controversial [2].

Since the beginning of vaccination against severe acute respiratory syndrome coronavirus 2 (SARS-CoV-2) in the global COVID-19 pandemic, few cases of adverse events associated with occurrence or deterioration of demyelinating CNS diseases such as multiple sclerosis (MS) have been published [3]. Regarding neuromyelitis optica spectrum disorders (NMOSD) two cases of manifestation with Aquaporin-4-antibody (AQP4) positive NMOSD after COVID-19 vaccination have been reported so far, one after application of the Oxford–AstraZeneca COVID-19 vaccine (AZD1222) and another case after vaccination with the Sputnik V COVID-19 vaccine (Gam-COVID-Vac), both viral vector vaccines [4, 5]. Furthermore, one case of occurrence of longitudinal extensive transverse myelitis (LETM) after the mRNA Moderna COVID-19 vaccine (mRNA-1273) has been reported, however due to absence of AQP4-antibodies and other diagnostic criteria, this patient does not fulfil NMOSD diagnostic criteria [6, 7].

Herein, we present a case of AQP4-antibody positive NMOSD presenting as severe LETM following first vaccination with the mRNA Pfizer–BioNTech COVID-19 vaccine (BNT162b2) in a female patient with suspected MS for over 40 years.

## Case presentation

A 68-year-old Caucasian woman presented with a rapidly progressive severe sensorimotor paraparesis a few days after having received the first mRNA COVID-19 vaccination (BNT162b2) in May 2021. The patient’s history showed the suspected diagnosis of a secondary progressive MS for over 40 years with unknown date of transition. After having suffered a severe relapse in the early 1980s the patient had lived with a residual mild paraparesis. The patient had never received a disease-modifying therapy (DMT). Relapses had been treated with intravenous corticosteroids. Regarding her vaccination history, the patient had regularly received the vaccines recommended by the German Standing Committee on Vaccination without any relevant side effects prior to the following events.

In April 2021, the patient first suffered a deterioration of the paraparesis shortly after having been vaccinated against tetanus and pneumococci. She was admitted to an external hospital. Spinal cord MRI showed signs of atrophy of the cervical and thoracic spinal cord without Gadolinium (GD)-enhancement. Cerebrospinal fluid (CSF) analysis did not show any abnormalities. Due to a suspected MS relapse the patient was treated with intravenous corticosteroids. Subsequently the symptoms receded and the patient reached prior level of disability.

**Fig. 1:**
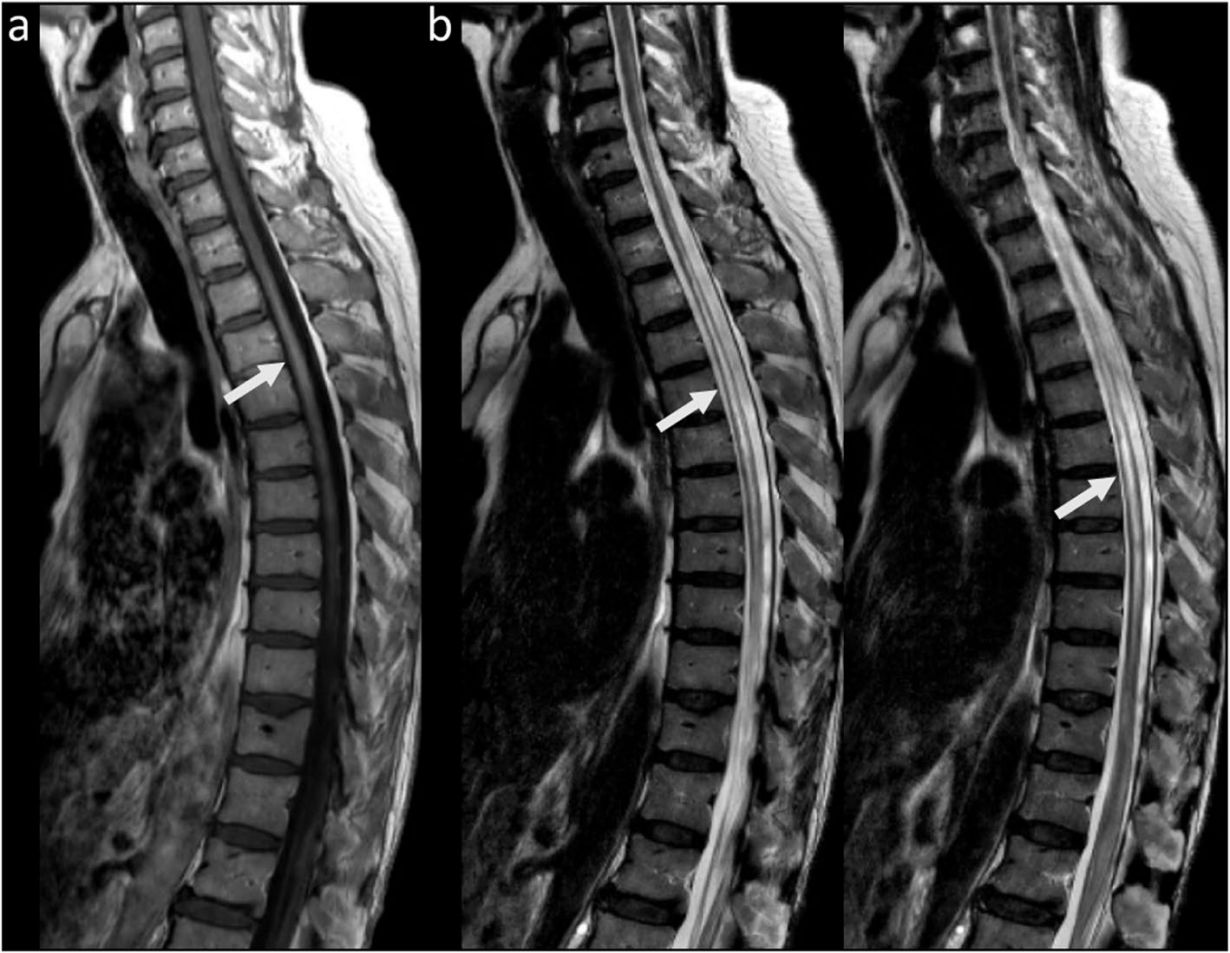
Spinal cord MRI (**a**) Sagittal T1-weighted + GD image; white arrow indicates GD-enhancement ranging from C3 to C5 (**b**) Sagittal T2-weighted images; white arrows indicate T2-signal alteration ranging from C4 to T10

On the 5th of May 2021 the patient received the first mRNA COVID-19 vaccination and 23 days later developed a severe exacerbation of the paraparesis with sensory level of T8 and inability to walk as well as loss of bladder and bowel control (EDSS 7.0). A new spinal cord MRI now showed T2-signal alteration ranging from C4 to T10 with GD-enhancement from C3 to C5 as sign of an acute LETM (Fig. 1). CSF analysis displayed pleocytosis of 340 cells/μl (51% lymphocytes, 49% granulocytes) and disturbance of the blood-brain-barrier with elevated protein levels of 2590 mg/L. There was no evidence of oligoclonal bands. Also, there was no evidence of bacterial or viral infection. Cell-based assay showed positive AQP4-antibodies in serum and CSF. The patient fulfilled diagnostic criteria for AQP4-antibody positive NMOSD.

Initial therapy with intravenous corticosteroids for five days was followed by seven cycles of plasmapheresis which led to a mild improvement of sensorimotor function (EDSS remaining at 7.0). Considering the highly active disease progression, DMT with eculizumab was initiated promptly. Since the patient showed deterioration of symptoms both times she was immunized, required meningococcal vaccination was not applied for safety reasons and prophylactic antibiotic therapy was initiated in the induction phase of eculizumab. The patient later decided not to receive the vaccination at all, hence therapy was changed to satralizumab. In the follow-up period of six months no new relapses have occurred. The patient also declined the second COVID-19 vaccination.

## Discussion

AQP4-antibody positive NMO represents a rare chronic inflammatory disorder of the CNS as a result of an autoantibody- and complement-mediated astrocytopathy due to high expression of the antigen AQP4 at the astrocytic endfeet, which is followed by secondary demyelination and often extensive axonal and neuronal damage, which primarily affects the spinal cord, optic nerves and brain stem areas [7]. Due to prognostic differences and differential response to diverse immunomodulatory treatments, with MS drugs mostly aggravating NMO disease activity, differential diagnosis of NMOSD and MS is crucial, in particular with regard to DMT initiation [8].

In this case the patient’s clinical presentation had been classified as MS in the early 1980s and not re-evaluated since, presumably as she had not suffered from a severe relapse until recently. Furthermore, the patient has never been treated with a DMT. Interestingly she already had suffered a mild exacerbation without any correlate in spinal cord imaging after having been vaccinated against tetanus and pneumococci, possibly indicating slight activation of the immune system. Following immunization with an mRNA COVID-19 vaccine the patient suffered from a severe disease exacerbation presenting as LETM which led to clinical re-evaluation and ultimately diagnosis of NMO due to detection of AQP4-autoantibodies in both serum and CSF.

The timeline of these events is highly suggestive for a potential triggering of so far self-contained autoimmune-mediated processes increased by the COVID-19 vaccination. Of note, causality between vaccination and relapse or manifestation of NMOSD have not been verified yet. Also, specific testing for serum autoantibodies against SARS-CoV-2 as a potential marker for the immune response to the COVID-19 vaccine has not been performed. Furthermore, it has to be taken into account that the patient received three types of vaccinations in a short period of time, so possible delayed effects of the first vaccinations or combined effects of an unspecific “bystander immune activation” as a consequence of these different vaccinations could be associated with the disease exacerbation observed in this particular case [9]. The patient’s history suggests that she suffered from a long-standing latent NMOSD misdiagnosed as MS that flared up after the vaccination. However, testing for AQP4-antibodies has not been performed prior to the severe relapse as proof of this hypothesis. Hence, the possibility that this patient actually did develop a new autoimmune disorder on basis of a recently inactive MS has to be acknowledged. However, one crucial factor that needs to be emphasized is the fact that this patient did not receive any DMT, especially not around the time of vaccination, which might have reduced the risk of disease exacerbation. So far, there have been no reports of disease exacerbation of properly treated NMOSD patients following COVID-19 vaccination, although post-vaccination manifestation or relapses of NMOSD have been reported before the COVID-19 pandemic [10, 11]. Since SARS-CoV-2-infection itself can also be associated with severe neurological disorders including demyelinating diseases of the CNS, the so far described rare cases of post-vaccination events should not lead to a change of vaccination policy or discouragement of patients [12].

## Conclusions

Taken together, this case adds evidence to the few reported cases of NMOSD manifestation upon COVID-19 vaccination and warrants to potentially avoid repetitive vaccinations against COVID-19 combined with other vaccinations with only a short in-between interval, if possible. Furthermore, effective treatment must be ensured in patients suffering from inflammatory CNS diseases before immunization. Finally, regarding the advances in diagnostic possibilities for neurologic disorders re-evaluation of diagnoses from the past should always be considered.

## Data Availability

The datasets used and/or analysed during the current study are available from the corresponding author on reasonable request.

## Abbreviations

AQP4: Aquaporin-4
CNS: Central nervous system
CSF: Cerebrospinal fluid
DMT: Disease-modifying therapy
EDSS: Expanded Disability Status Scale
GD: Gadolinium
HPV: Human papilloma virus
LETM: Longitudinal extensive transverse myelitis
MS: Multiple sclerosis
NMOSD: Neuromyelitis optica spectrum disorder
SARS-CoV-2: Severe acute respiratory syndrome coronavirus 2
